# Impact of livestock interventions on maternal and child nutrition outcomes in Africa: A systematic review and meta-analysis protocol

**DOI:** 10.12688/aasopenres.13150.2

**Published:** 2021-10-01

**Authors:** Josphat Muema, Julius Oyugi, Zipporah Bukania, Mutono Nyamai, Christine Jost, Tewoldeberhan Daniel, Joseph Njuguna, Samuel Mwangi Thumbi

**Affiliations:** 1 Institute of Tropical and Infectious Diseases, University of Nairobi, Nairobi, Kenya; 2 Washington State University, Global Health Program - Kenya, Nairobi, Kenya; 3 Centre for Public Health Research, Kenya Medical Research Institute, Nairobi, Kenya; 4 Wangari Maathai Institute of Peace and Environmental studies, University of Nairobi, Nairobi, Kenya; 5 United States Agency for International Development’s office for U.S Disaster Assistance, Washington, DC, USA; 6 United Nations Children’s Fund, Nairobi, Kenya; 7 Food and Agriculture Organization of the United Nations, Nairobi, Kenya; 8 Centre for Global Health Research, Kenya Medical Research Institute,, Nairobi, Kenya; 9 Paul G. Allen School for Global Animal Health, Washington State University, Pullman, USA

**Keywords:** Livestock interventions, Malnutrition, Under-five children, Systematic review, Africa

## Abstract

The challenge of undernutrition (stunting and wasting) still remains a major health concern in children below 5 years of age in Africa, with the continent accounting for more than one third of all stunted children and more than one quarter of all wasted children globally. Despite the growing evidence on the role of agriculture interventions in improving nutrition, empirical evidence on the impact of livestock intervention on nutrition in Africa is scant.

This review is aimed at determining whether livestock interventions are effective in reducing undernutrition in children below five years of age and in pregnant and lactating women in Africa. The review will be conducted according to PRISMA guidelines. Major electronic databases will be searched and complemented with grey and non-indexed literature from google and google scholar, and expert consultation for additional articles and reports. PICO criteria will be used while employing search strategies including MeSH, Boolean search operators and truncation/wildcard symbol to narrow or broaden the search. Articles on effect of livestock interventions on maternal and child nutrition conducted in Africa that meet the set inclusion criteria will be included in the review after critical appraisal by two independent reviewers. A standardized form will be used to extract data from included studies. The extracted data will be summarized and synthesized both qualitatively and quantitatively and key outcomes presented. Evidence generated from the systematic review and meta-analysis will be important for guiding nutrition sensitive livestock interventions and policies on nutrition programming, specifically on how to leverage on livestock interventions to reduce the burden of undernutrition.

## Introduction

Undernutrition (stunting, wasting and underweight) remains a serious public health concern in Africa ^1^. Globally, the continent accounted for more than one third of stunted children (58.7 million, 39%) and more than one quarter of wasted children (13.8 million, 27%) in 2018 ^2, 3^. To combat and address the challenge of undernutrition and contribute to the progress of attaining Sustainable Development Goal 2 of ending hunger and all forms of malnutrition, holistic, multifaceted strategies employing both nutrition specific and nutrition sensitive interventions are required ^4, 5^. Additionally, more efforts are needed in building more resilient, equitable and sustainable food systems for improved nutrition ^6^

Agriculture including livestock plays a key role as a source of food and nutrition security and livelihoods for a majority of rural households in sub-Saharan Africa ^7^. In the last two decades, several reviews have been conducted to assess the contribution/impact of general agriculture interventions (home gardening for fruits and vegetables, aquaculture, livestock production, cash crops and biofortified crops) on nutrition ^8– 18^. These reviews have documented the growing evidence on the role of agriculture interventions in improving nutrition and identified some of the pathways through which agriculture interventions can contribute to nutrition. Animal-source foods (ASF) are a rich source of bioavailable nutrients that play an important role in reducing risk of protein malnutrition ^19, 20^. In the context of arid and semi-arid areas with limited potential for crop agriculture, the role of livestock and ASF in supporting the livelihood and nutrition of pastoralist communities is especially critical.

The impact pathways through which livestock interventions may influence human nutrition include: (1) Increased production and consumption of animal source foods and hence dietary diversity at household and individual level (2) Increased household level income through sale of livestock products which in turn translates into increased access to dietary diversity ^18, 21^. Livestock interventions such as dairy programs, small livestock rearing, backyard poultry production, breed improvement, fisheries, livestock transfer programs, livestock feeds improvement and livestock value chains programs have a potential to positively influence improved dietary diversity at household level and possibly impacting the individual nutritional outcomes. However, empirical data on the net contribution of livestock intervention on nutrition in Africa is scant.

This review is aimed at collating, synthesizing and documenting all available evidence on the linkages between livestock interventions and nutrition outcomes in Africa. Evidence generated from the systematic review and meta-analysis will be important for guiding nutrition sensitive livestock interventions and policies on nutrition programming, specifically on how to leverage on livestock interventions to reduce the burden of undernutrition.

### Review question

Are livestock interventions effective in reducing undernutrition in children below five years of age and in pregnant and lactating women in Africa? 

**Objectives of this review are to**

1. Assess the available evidence on impact of livestock interventions on maternal and child nutrition outcomes in Africa and identify data gaps
2. Determine the characteristics (design and implementation strategies) of livestock interventions that improve nutrition outcomes
3. Estimate the pooled effect of livestock interventions to improve nutrition outcomes
4. Evaluate the type of livestock interventions more effective in improving nutrition outcomes

## Methods

The systematic review will be conducted following the guidelines suggested in the PRISMA (Preferred Reporting Items for Systematic Reviews and Meta-Analysis) statement ^22, 23^. The review protocol has been registered in the international prospective register of systematic reviews (PROSPERO), protocol registration number CRD42020203843 on 14 ^th^ September 2020.

### Definitions

Livestock interventions – all livestock related interventions or programmes with an objective of increasing production diversity, access and consumption of animal source foods (ASFs) and income generation to the households. Such interventions include provision of livestock feed, provision of animal health care, provision of water, provision of shelter, and training/extension services

Livestock – all domesticated animals such as cattle, camels, goats, sheep, pigs, other small ruminants, poultry/chicken, fish and bees

### Literature search and study selection

Major electronic databases including PubMed, Scopus and Web of Science will be searched by two independent reviewers to identify relevant peer-reviewed publications and reports. All the reference lists of all papers identified through the database searches and relevant papers and reports considered will be reviewed and “forward citation” tool in Google Scholar will be applied to find papers that cited these studies to complement the search. Reference lists of previous systematic reviews conducted on similar study themes will also be reviewed. Experts in this field and study investigators will also be consulted for any additional papers or reports which may not have been captured through the online search.

Search strategy will be based on key words formulated according to the population/patient/problem, intervention/indicator/exposure, comparison/control, outcome (PICO) format. These key words will be generated through a preliminary general search in major electronic databases to identify most used key words in the publications. Medical Subject Headings (MeSH) terms will be used to identify potential key wards and choose appropriate terms. Boolean operators’ terms “AND”, “OR” and “NOT” will be used to connect the search terms to either narrow or broaden the search. Truncation/wildcard symbol (*) will also be used for words where variations may be possible ( Table 1).

**Table 1.** Key words and search terms to be used in database searches.

Indicator	Description
Population	Child OR Infant OR Pediatric OR “young adult” OR Preschool OR Pregnant OR Woman OR Women OR Lactating OR Breastfeeding OR Adolescent OR toddler
Intervention	Trial OR Programme OR Intervention OR Experiment OR Supplementation OR Implementation OR Feed OR Consumption OR “Livestock production” OR “livestock ownership” OR Pastoral OR Livestock OR Cattle OR Camel OR Goat OR Sheep OR Small ruminant OR Poultry OR Chicken OR Fish OR Aquaculture OR fish pod OR Pig OR Meat OR Beef OR mutton OR Pork OR dairy OR egg OR honey OR “animal source food” OR “animal products” OR “foods of animal origin” OR “nutrition sensitive agriculture” OR value chain OR Beekeeping OR “animal health care” OR water OR shelter OR training Or extension services
Outcome	Nutrition OR nutrition status OR nutrition outcome OR Growth OR Linear Growth OR Malnutrition OR Undernutrition OR Stunting OR Wasting OR underweight OR Micronutrient OR micronutrient status OR anemia OR hemoglobin OR hemoglobin OR folate OR vitamin OR Vitamin A OR Vitamin B12 OR iron OR Ferritin OR zinc OR calcium OR MUAC OR anthropometric OR Height-for-age OR Weight-for-height OR Weight-for-age OR dietary diversity
Geographical location	Developing Countries OR Africa OR Africa, Northern OR Africa South of the Sahara OR Sub-Saharan Africa OR Africa, Central OR Africa, Eastern OR Africa, Southern OR Africa, Western OR Algeria OR Angola OR Benin OR Botswana OR Burkina Faso OR Burundi OR Cameroon OR Cape Verde OR Central African Republic OR Chad OR Comoros OR Congo OR “Cote d’Ivoire” OR Djibouti OR “Democratic Republic of the Congo” OR Egypt OR Eritrea OR Ethiopia OR Gabon OR Gambia OR Ghana OR Guinea OR Guinea-Bissau OR Kenya OR Lesotho OR Liberia OR Libya OR Madagascar OR Malawi OR Mali OR Mauritania OR Mauritius OR Morocco OR Mozambique OR Namibia OR Niger OR Nigeria OR Rwanda OR Senegal OR Seychelles OR Sierra Leone OR Somalia OR South Africa OR Sudan OR Swaziland OR Tanzania OR Togo OR Tunisia OR Uganda OR Zambia OR Zimbabwe

MUAC – mid-upper arm circumference

### Inclusion and exclusion criteria

Studies will be screened against a set inclusion and exclusion criteria to determine and assess their relevance for inclusion in the systematic review ( Table 2). Studies published in any date up to the time of conducting the search and which meet the inclusion criteria will be eligible for inclusion.

**Table 2.** Inclusion and exclusion criteria to be used to assess study eligibility.

Criteria	Include	Exclude
Location	Studies conducted in Africa	Studies conducted in other continents
Population	Children below 5 years, OR pregnant women OR Lactating women	
Intervention	Livestock interventions contributing to production and consumption of animal source foods (milk, meat, eggs and fish) and livestock value chains	Crop agriculture Biofortification Home gardening Irrigation programs
Outcome	Nutrition outcomes including; anthropometry (weight-for-age z-score, height-for-age z-score, weigh-for-height z-score, MUAC, micronutrient status and health related outcomes	Health outcomes not directly related to nutrition
Publication date	Any date	
Publication type	Peer reviewed articles and online reports	Unpublished reports
Study designs	Experimental, quasi-experimental and observational studies, cross-sectional longitudinal intervention-control comparisons and randomized field trials	Literature reviews
Publication language	English	Other languages

MUAC – mid-upper arm circumference

### Data management

The search results will be uploaded to Rayyan QCRI a web and mobile app for systematic reviews that facilitates collaboration among reviewers during the study selection process. All duplicates will be removed prior to screening using the Rayyan QCRI platform. Screening questions will be developed and tested based on the inclusion and exclusion criteria prior to the start of the screening process.

A two-stage screening process will be employed in all the retrieved articles from the database searches;

i. Titles/abstracts will be screened by two independent reviewers for relevance to the review question
ii. Full texts of possible relevant articles will be reviewed by two independent reviewers to ascertain if the methods used in the studies selected at stage one adheres to the set methodological standards for the review and exclude those that do not meet the criteria. Any disagreement between the two reviewers over the eligibility of any study will be decided through discussion with a third reviewer and consensus reached.

### Data abstraction and synthesis

For those articles found relevant after full text review, data will be extracted using a pre-prepared excel spreadsheet template (extended data ^24^). Variables to be extracted are described in Table 3

**Table 3.** Data abstraction variables for the full text articles included in the review.

#	Variable	Description
1	Author(s)	The lead author of the study
2	Year	The year the study was published
3	Study geographical location	The country study was done
4	Title	Full title of the study
5	Publication type	Peer reviewed journal article, report or student thesis
6	Study design	Experimental (controlled trials), or observational (Cohort studies, case-control studies, and cross-sectional studies)
7	Study participants	Study population characteristics
8	Overall sample size	Number of study participants included in the study
9	Exposure measure	For observational studies
10	Intervention type	For experimental studies
11 12	Outcome measured Intermediate outcome measured	Micronutrient status and anthropometry – height-for-age(stunting), weight-for- height(wasting) and weight-for-age(underweight) Dietary diversity, income and morbidity
13	Effect of intervention on nutrition	The difference in nutrition outcomes between intervention and control groups
14	Statistical significance	Measures of statistical significance used and their corresponding values
15 16	Study findings Study limitations	Summary of key study findings
17	Conclusion	
18	Reference	

The data extracted will be summarized and synthesized both qualitatively and quantitatively and key outcomes presented. For the qualitative data, a summary of key outcomes will be provided using Excel. For the quantitative synthesis statistical software RevMan 5.1 will be used. The primary outcome measure will be the nutrition status of children below five years and pregnant and lactating women and will be measured through micronutrient status or anthropometry (stunting, wasting and underweight). The intermediate outcome measure will be dietary diversity, incomes and morbidity.

Homogeneity in reporting metrics of the included studies will be assessed. If a sufficient number of studies reporting on effect of livestock interventions on nutrition are identified and there is consistency in reporting metrics, a meta-analysis will be conducted on this subset of studies. Pooled/summary effect estimate of livestock interventions will be calculated using relative risk ratios (RR) and their corresponding 95% confidence intervals (CI). The percentage of variations across the studies and their impact on the meta-analysis will be quantified by calculating and reporting the statistical measure of heterogeneity (I ^2^ statistic) and obtaining a summary estimate of the effect of livestock interventions on nutrition. For the studies included in the quantitative synthesis, a fixed and random effect models will be used to calculate the RR and 95% CI based on the level of heterogeneity (I ^2^ statistic) ^25, 26^. If high levels of heterogeneity are detected ((I ^2^ >=50% or P <0.1) we will perform a sub-group analysis to determine and explain the source of heterogeneity. If heterogeneity is observed in the sub-group analysis, a meta-regression will be conducted. 

### Validity/risk of bias assessment

Individual studies will be assessed for both internal and external validity. The Grades of Recommendations, Assessment, Development and Evaluation (GRADE) guidelines ^27^ will be used to assess study validity/risk of bias. Studies will be scored as either low, medium and high quality based on five criteria; counterfactual analysis, sample size and power calculations, nutrition outcome assessment, intermediate outcome assessment and confounding bias assessment. Overall assessment of risk of bias for each study will be determined through weighted judgement of the established criteria.

### Presentation of results

Data summarized and synthesized using qualitative methods will be presented inform of summary tables of key outcomes together with a narrative description of the studies using excel.

For the quantitative analysis (meta-analysis) statistical software RevMan 5.1 will be used for analysis and the output will be presented graphically using a forest plot indicating point estimate and 95% confidence interval of observed effect for each individual study together with summary estimate and its confidence interval.

### Dissemination of information

The review findings will be disseminated through open access publication of the results, as well as dissemination in seminars and workshops

### Study status

We are currently piloting the study selection process

## Data availability

### Underlying data

No data are associated with this article

### Extended data

Open Science Framework: Impact of livestock interventions on maternal and child nutrition outcomes in Africa: A systematic review and meta-analysis protocol. https://doi.org/10.17605/OSF.IO/7GMHC ^24^

This project contains the following extended data:

- Data Abstraction sheet.xlsx (Study data extraction sheet)

### Reporting guidelines

Open Science Framework: PRISMA-P checklist for ‘Impact of livestock interventions on maternal and child nutrition outcomes in Africa: A systematic review and meta-analysis protocol’ https://doi.org/10.17605/OSF.IO/7GMHC ^24^

Data are available under the terms of the Creative Commons Zero "No rights reserved" data waiver (CC0 1.0 Public domain dedication).
