# Supplementary material for: Impact of livestock interventions on maternal and child nutrition outcomes in Africa: A systematic review and meta-analysis protocol
Source: AAS Open Res. 2021 Oct 1;4:1. Originally published 2021 Jan 8. [Version 2] doi: 10.12688/aasopenres.13150.2 (PMC8552048; doi:10.12688/aasopenres.13150.2)
Supplement: Peer review history

# Peer review: Reviewer response for version 2

**DOI:** 10.21956/aasopenres.14436.r28923

**Published:** 2021-10-27

**Authors:** Willy Kiboi

**Affiliations:** 1 School of Nursing and Public Health, Chuka University, Chuka, Kenya

The revisions have satisfactorily addressed the concerns.

Is the study design appropriate for the research question?

Yes

Is the rationale for, and objectives of, the study clearly described?

Yes

Are sufficient details of the methods provided to allow replication by others?

Partly

Are the datasets clearly presented in a useable and accessible format?

Not applicable

Reviewer Expertise:

Maternal and Child Nutrition

I confirm that I have read this submission and believe that I have an appropriate level of expertise to confirm that it is of an acceptable scientific standard.

# Peer review: Reviewer response for version 2

**DOI:** 10.21956/aasopenres.14436.r28924

**Published:** 2021-10-04

**Authors:** Nazia Binte Ali

**Affiliations:** 1 Maternal and Child Health Division (MCHD), International Centre for Diarrhoeal Disease Research, Bangladesh (icddr,b), Dhaka, Bangladesh

The article is now suitable for indexing.

Is the study design appropriate for the research question?

Yes

Is the rationale for, and objectives of, the study clearly described?

Yes

Are sufficient details of the methods provided to allow replication by others?

Partly

Are the datasets clearly presented in a useable and accessible format?

Not applicable

Reviewer Expertise:

Maternal and child health and nutrition in the context of low and middle income countries

I confirm that I have read this submission and believe that I have an appropriate level of expertise to confirm that it is of an acceptable scientific standard.

# Peer review: Reviewer response for version 1

**DOI:** 10.21956/aasopenres.14251.r28797

**Published:** 2021-08-27

**Authors:** Willy Kiboi

**Affiliations:** 1 School of Nursing and Public Health, Chuka University, Chuka, Kenya

The paper is generally well written. The methodology used is appropriate but requires some improvements. The following comments may help improve the paper;

- In the abstract and the introduction sections, the authors may wish to explain why they are excluding underweight children, when in the methodology they state that they will be considered (Table 2).
- Let the authors write all abbreviations in full in their first use.
- The authors talk about maternal and child nutrition outcomes in the topic, and yet the aspect of maternal nutrition (pregnant and lactating mothers) is relatively silent in the whole paper.
- In the second paragraph of the introduction section, the authors mention that several reviews have been conducted to assess the contribution/impact of general agriculture interventions including livestock production on nutrition. Could the authors explain why the review will be necessary if such data exists?
- In the objectives of the review, it’s not clear what the authors mean by characteristics of livestock interventions (objective 2).
- The authors may consider explaining further how the study selection will be conducted e.g. how will duplicate and irrelevant articles be excluded.
- In the study designs to be included, the authors may wish to redefine the designs. For example, quasi-experimental designs are part of experimental studies. I also find the term “observational studies” to be a broad term when explaining a research design. I suggest that the authors be more specific on the research designs that they wish to include in their review.
- Below Table 2 is the word MUAC (mid-upper arm circumference) which seems to be misplaced. If it is an explanation of the abbreviation, then the authors may wish to have a section with a list of all used abbreviations and their explanations.
- In the data management section, the authors state that only articles where there is concurrence between the two reviewers will be included in the review. Inconsistent with that statement, the subsequent statement says that the disagreement between the two reviewers will be discussed with a third reviewer. The researchers may wish to relook at those statements.
- Furthermore, the authors mention that the primary outcome measure will be the nutrition status of children below five years and women of childbearing age. I find this to have departed from the earlier stated study population (pregnant and lactating women). Women of reproductive age may include all women aged 15-49 as defined by WHO.
- In the study's heterogeneity assessment, the authors have some contradictory statements. For example, the authors state that “if high levels of heterogeneity are detected, we will perform a subgroup analysis to determine and explain the sources of heterogeneity”. In the subsequent statement the authors state that “If heterogeneity is substantial, a meta-analysis will not be performed”.
- The authors may consider conducting a meta-regression if heterogeneity is further observed in the subgroup analysis.

Is the study design appropriate for the research question?

Yes

Is the rationale for, and objectives of, the study clearly described?

Yes

Are sufficient details of the methods provided to allow replication by others?

Partly

Are the datasets clearly presented in a useable and accessible format?

Not applicable

Reviewer Expertise:

Maternal and Child Nutrition

I confirm that I have read this submission and believe that I have an appropriate level of expertise to confirm that it is of an acceptable scientific standard, however I have significant reservations, as outlined above.

# Response

**Published:** 2021-09-22

**Authors:** Josphat Muema

**Affiliations:** Washington State University, Kenya

Thank you for your insightful comments. We have carefully addressed each of the comments as detailed below.

*In the abstract and the introduction sections, the authors may wish to explain why they are excluding underweight children, when in the methodology they state that they will be considered (Table 2).*

- ​​​​​​This has been reviewed and underweight children has been included

​​​​​​ *Let the authors write all abbreviations in full in their first use.*

- ​​​​​​All abbreviations have been written in full in their first use

*​​​​​​The authors talk about maternal and child nutrition outcomes in the topic, and yet the aspect of maternal nutrition (pregnant and lactating mothers) is relatively silent in the whole paper.*

- ​​​​​​This has been clarified. Studies evaluating maternal nutrition outcomes for pregnant and lactating mothers will be included in the review. 

*In the second paragraph of the introduction section, the authors mention that several reviews have been conducted to assess the contribution/impact of general agriculture interventions including livestock production on nutrition. Could the authors explain why the review will be necessary if such data exists?*

- ​​​​​​Most of the reviews have focused on crop agriculture. The few livestock reviews have not been done specifically for Africa and have not focused on their impact on nutrition for children under five years and pregnant and lactating women. Our review is aimed at providing evidence for the impact of livestock interventions on nutrition outcomes for children under five years and pregnant and lactating women who are among the most nutritionally vulnerable groups specifically in Africa setting.

*In the objectives of the review, it’s not clear what the authors mean by characteristics of livestock interventions (objective 2).*

- This has been clarified, it will be in terms of design and implementation strategies.

*The authors may consider explaining further how the study selection will be conducted e.g. how will duplicate and irrelevant articles be excluded.*

- This has been clarified. The duplicates and irrelevant articles will be excluded using the Rayyan QCRI application which is a web and mobile phone app for systematic reviews

*In the study designs to be included, the authors may wish to redefine the designs. For example, quasi-experimental designs are part of experimental studies. I also find the term “observational studies” to be a broad term when explaining a research design. I suggest that the authors be more specific on the research designs that they wish to include in their review.*

- For clarity quasi-experimental designs has been removed and examples of observational studies to be considered provided including cohort studies, case-control studies, and cross-sectional studies

*Below Table 2 is the word MUAC (mid-upper arm circumference) which seems to be misplaced. If it is an explanation of the abbreviation, then the authors may wish to have a section with a list of all used abbreviations and their explanations.*

- ​​​​​​All other Abbreviations have been defined in the text except MUAC which has not been used in the text hence defined under the table where it is used.

*​​​​​​In the data management section, the authors state that only articles where there is concurrence between the two reviewers will be included in the review. Inconsistent with that statement, the subsequent statement says that the disagreement between the two reviewers will be discussed with a third reviewer. The researchers may wish to relook at those statements.*

- ​​​​​​This has been clarified. Studies will be examined independently by two reviewers. Studies that meet inclusion criteria will pass for full text review for eligibility by two reviewers. Any disagreement between them over the eligibility of any study will be decided through discussion with a third reviewer.

*Furthermore, the authors mention that the primary outcome measure will be the nutrition status of children below five years and women of childbearing age. I find this to have departed from the earlier stated study population (pregnant and lactating women). Women of reproductive age may include all women aged 15-49 as defined by WHO.*

- ​​​​​​For consistency, this has been reviewed and clarified to be pregnant and lactating women

*​​​​​​In the study's heterogeneity assessment, the authors have some contradictory statements. For example, the authors state that “if high levels of heterogeneity are detected, we will perform a subgroup analysis to determine and explain the sources of heterogeneity”. In the subsequent statement the authors state that “If heterogeneity is substantial, a meta-analysis will not be performed”.*

- This has been clarified and redundancies in the statement removed. Sub-group analysis will be performed for heterogeneity assessment.

*The authors may consider conducting a meta-regression if heterogeneity is further observed in the subgroup analysis.*

- We agree with the reviewer that it will be beneficial to conduct a meta-regression if heterogeneity is observed in the subgroup analysis. This has been included in the protocol

# Peer review: Reviewer response for version 1

**DOI:** 10.21956/aasopenres.14251.r28513

**Published:** 2021-04-12

**Authors:** Nazia Binte Ali

**Affiliations:** 1 Maternal and Child Health Division (MCHD), International Centre for Diarrhoeal Disease Research, Bangladesh (icddr,b), Dhaka, Bangladesh

Many congratulations to the authors for selecting such a timely and important topic for review. The evidence generated by this paper will be important to the policymakers and researchers to identify effective modalities of livestock interventions for improving maternal and child nutrition statuses. Overall the paper is well written with well-established rationales. I have few comments on the methodology which may improve the manuscript;

1. For selecting keywords for literature search, I would suggest including MeSH terms. This will help identify all the potential keywords and will reduce the chance of missing.
2. Although it was mentioned in table 2 that any publication date will be considered, it is better to cite that in the method write up.
3. Table 2 is contradictory to the method write-up in terms of literature exclusion criteria. In the table exclusion criteria it was mentioned that unpublished reports will be excluded while in the method section it was mentioned that grey literature will be included. I suggest to clarify the inconsistencies.
4. Table 2: mentioned that feasibility studies will be excluded from this review. Feasibility studies encompass a wide range starting from pilot projects to effectiveness studies. What was the rationale behind this exclusion?
5. There is mention about using multiple search engines. In that case, the first step will be the identification and removal of duplicate literature. I suggest including duplicate identification and removal methods.

Is the study design appropriate for the research question?

Yes

Is the rationale for, and objectives of, the study clearly described?

Yes

Are sufficient details of the methods provided to allow replication by others?

Partly

Are the datasets clearly presented in a useable and accessible format?

Not applicable

Reviewer Expertise:

Maternal and child health and nutrition in the context of low and middle income countries

I confirm that I have read this submission and believe that I have an appropriate level of expertise to confirm that it is of an acceptable scientific standard, however I have significant reservations, as outlined above.

# Response

**Published:** 2021-09-22

**Authors:** Josphat Muema

**Affiliations:** Washington State University, Kenya

Thank you for your comments which have significantly improved our article. We have carefully addressed each of the comments as follows

*For selecting keywords for literature search, I would suggest including MeSH terms. This will help identify all the potential keywords and will reduce the chance of missing.*

- We agree with the reviewer and Medical Subject Headings (MeSH) has been included as part of the search strategy.

*​​​​​​Although it was mentioned in table 2 that any publication date will be considered, it is better to cite that in the method write up*

- This has been included in the methods as part of the inclusion criteria.

*​​​​​Table 2 is contradictory to the method write-up in terms of literature exclusion criteria. In the table exclusion criteria, it was mentioned that unpublished reports will be excluded while in the method section it was mentioned that grey literature will be included. I suggest clarifying the inconsistencies.*

- We agree with the reviewer, and for consistency we have deleted the section in the text indicating inclusion of grey literature.

*Table 2: mentioned that feasibility studies will be excluded from this review. Feasibility studies encompass a wide range starting from pilot projects to effectiveness studies. What was the rationale behind this exclusion?*

- ​​​​​​Majority of pilot and feasibility studies are non-randomized and do not include power calculations to test hypothesis. However, some are conducted within standards hence could provide vital evidence. Consequently, in this review feasibility studies will be evaluated against the set criteria and those that meet the set criteria will be included

*There is mention about using multiple search engines. In that case, the first step will be the identification and removal of duplicate literature. I suggest including duplicate identification and removal methods.*

- Duplicates will be removed using the Rayyan QCRI web and mobile app for systematic reviews.

​​​​​​
